# A novel electrochemical immunosensor based on biomaterials for detecting carcinoembryonic antigen biomarker in serum samples

**DOI:** 10.1038/s41598-025-09547-1

**Published:** 2025-07-14

**Authors:** Sajedeh Sobhanparast, Payam Shahbazi-Derakhshi, Jafar Soleymani, Amir Amiri-Sadeghan, Alireza Herischi, Nader Chaparzadeh, Younes Aftabi

**Affiliations:** 1 Department of Biology, Azarbaijan Shahid Madani University, Tabriz, Iran; 2 Tuberculosis and Lung Diseases Research Center, Tabriz University of Medical Sciences, Tabriz, Iran; 3 Liver and Gastrointestinal Diseases Research Center, Tabriz University of Medical Sciences, Tabriz, Iran; 4 Pharmaceutical Analysis Research Center, Tabriz University of Medical Sciences, Tabriz, Iran

**Keywords:** Carcinoembryonic antigene, Label-free, Electrochemical, Biomaterials, ELISA, Serum samples, Cancer, Analytical chemistry

## Abstract

A new label-free electrochemical immunosensor was developed to detect carcinoembryonic antigen (CEA), a key diagnostic biomarker for various cancers. The surface of a glassy carbon electrode (GCE) was modified using a layer-by-layer assembly method incorporating sodium alginate (SA), gold nanoparticles (AuNPs), and gamma-manganese dioxide/chitosan (γ.MnO₂-CS). This modification enhanced the functional surface area and electrode conductivity, thereby improving the electrochemical response and sensitivity for CEA detection. Methods including cyclic voltammetry (CV) and differential pulse voltammetry (DPV) were used to evaluate the immunosensor`s performance.The sensor quantified CEA concentrations in serum samples by monitoring current variations at the oxidation peak, resulting from the formation of CEA antibody–CEA antigen complexes on the electrode surface. The sensor exhibited a linear response to CEA concentrations ranging from 10 fg/mL to 0.1 µg/mL, with a limit of detection (LOD) of 9.57 fg/mL and a limit of quantification (LOQ) of 31.6 fg/mL. The morphology and structural properties of the synthesized γ.MnO₂-CS nanocomposite and the modified electrode surface were characterized utilizing field-emission scanning electron microscopy (FE-SEM), Brunauer–Emmett–Teller (BET) and Barrett-Joyner-Halenda (BJH), X-ray diffraction (XRD), energy-dispersive X-ray spectroscopy (EDX), atomic force microscopy (AFM), dynamic light scattering (DLS), zeta potential analysis, and Fourier transform infrared (FTIR) spectroscopy. The results highlight the potential clinical applicability of this electrochemical immunosensor for detecting CEA levels in human serum samples.

**Supplementary Information:**

The online version contains supplementary material available at 10.1038/s41598-025-09547-1.

## Introduction

The immunoglobulin (Ig) superfamily consists of the carcinoembryonic antigen (CEA) family, which includes various glycoproteins, exhibiting significant variation among mammalian species. This family is primarily categorized into two main groups of pregnancy-specific glycoproteins (PSGs) and CEA-related cell adhesion molecules (CEACAMs), the latter being exclusively expressed in trophoblasts^1^. During fetal development, CEACAM5 (commonly known as CEA) is produced in gastrointestinal tissues. Due to the lack of its production after birth, healthy adults typically have very low concentrations of CEA in their blood, with an average concentration of 2–4 ng/mL^2,3^. However, elevated serum CEA levels have been detected in various malignancies, indicating its potential as a tumor biomarker in clinical settings.

As a key tumor marker, accurate detection of CEA is essential for differential diagnosis, disease monitoring, and treatment evaluation^4^. Conventional CEA detection methods primarily rely on immunoassays like chemiluminescent immunoassay (CLIA), radioimmunoassay (RIA) and enzyme-linked immunosorbent assay (ELISA)^5^. These methods, while widely used, require specialized equipment, trained personnel, and are often time-consuming. ELISA involves antigen-antibody complex formation on a solid surface, generating a quantifiable signal. However, it requires multiple washing steps, making it labor-intensive^6,7^. CLIA employs luminescent molecules for detection but has high operational costs and limited applicability for antigen quantification^8^. RIA uses radiolabeled antigens, which introduce potential health risks due to radiation exposure^7,9^. Beyond immunoassays, other high-sensitivity detection techniques—such as microscopy, cell culture, and molecular biology-based methods—exist but are time-intensive and unsuitable for rapid diagnostics^10,11^.

To address these limitations, biosensors have emerged as rapid, sensitive, and inexpensive tools for CEA quantification^12^. Biosensors integrate biological recognition components (e.g., enzymes, antibodies (Ab), DNA/RNA, or cells) with a physicochemical transducer, converting biological interactions into measurable signals^13–15^. Based on the type of biotransducer, biosensors can be classified into optical, electrochemical, piezoelectric, and thermometric types. Among them, electrochemical sensors become prominent because of their high sensitivity, specificity, portability, and ease of use^16^. These devices leverage the specific antigen (Ag)-Ab interaction, converting the biochemical signal into an electrochemical response.

Electrochemical immunosensors can be categorized depending on their detection mechanism, comprising amperometric, voltammetric, potentiometric, and conductometric approaches. They are further divided into label-free and labeled methods. Label-free immunosensors detect analytes directly through biochemical interactions on the sensor surface^17,18^. Labeled immunosensors employ tagged analytes (e.g., radioisotopes or fluorescent markers) to generate a detectable signal^19^. A key challenge in electrochemical sensing is surface modification, where the bioreceptor (Ab) is immobilized onto the electrode surface. The electrode material must be highly conductive and biocompatible while minimizing non-specific interactions to ensure optimal sensor performance^20^.

The incorporation of nano-sized materials has significantly enhanced the sensitivity, stability, and electrical properties of electrochemical-based biosensing probes^21–23^. For example, polysaccharide-based biomaterials have been widely utilized due to their chemical functionality, biocompatibility, and ability to form 3D electrode structures. These materials provide a porous, high-surface-area scaffold for enhanced analyte capture, improving signal transduction and electron transfer kinetics. Nanocomposites composed of sodium alginate (SA), gold nanoparticles (AuNPs), and gamma-manganese dioxide/chitosan (γ.MnO₂-CS) are particularly promising for electrochemical sensor applications^14,24,25^. SA is a biodegradable macromolecule derived from seaweed, offering stability, sensitivity, and a matrix for immobilizing Abs or Ags^26,27^. AuNPs exhibit excellent conductivity and enable stable biomolecule immobilization, significantly improving electron transfer^28^. CS serves as a biocompatible and 3D structure for retaning MnO₂ and then immobilizing Ab^29,30^. MnO₂, known for its catalytic properties, affordability, and environmental compatibility, enhances electrode sensitivity and surface area when integrated into hybrid nanocomposites^31–33^. Also, vacant orbitals of the MnO₂ can be used for the anchoring of CEA antibodies.

In this investigation, a simple, cost-effective, and label-free immunosening platformhas been reported for CEA detection in serum samples. The sensor fabrication involved immobilizing anti-CEA (Ab) onto a modified glassy carbon electrode (GCE) with γ.MnO₂-CS/AuNPs/SA nanomaterials. The sensor’s electrochemical performance was evaluated using differential pulse voltammetry (DPV) and cyclic voltammetry (CV) to astudy the redox properties, electroactive molecule behavior, and sensitivity. These techniques effectively reduced charging current interference, enhancing the precision of electrode reaction analysis. A ratiometric approach was employed to minimize matrix effects and sample interference, ensuring more accurate and reliable quantification of CEA in complex biological samples. This study presents a novel electrochemical immunosensor incorporating functional nanomaterials to improve CEA detection sensitivity and selectivity. The developed sensor demonstrates strong potential for clinical applications, offering a rapid, cost-effective, and highly sensitive alternative to conventional immunoassays.

## Materials, instruments, and methods

### Materials

Bovine serum albumin (BSA) was purchased from DNA Biotech (Iran). CEA was purchased from the company bioMérieux SA (France). Anti-CEA was received as a gift from Pishtazteb (Iran). Potassium permanganate (KMnO_4_), SA, CS, potassium ferricyanide (K_3_Fe(CN)_6_), and potassium ferrocyanide (K_4_Fe(CN)_6_) were gotten from Sigma-Aldrich (Germany). Phosphate-buffer (PB) solution (50 mM) was prepared using NaH_2_PO_4_ and Na_2_HPO_4_ salt at pH 7.5. A 2.5 mM solution of SA was prepared by dissolving in PB. AuNPs solution was prepared at a concentration of 250 µM. CS was dissolved in a mixture of ethanol (Merck, Germany) and water.

### Instruments

The biosensing process and electrochemical analysis were performed using an AUTOLAB galvanostat/potentiostat system. Three distinct electrodes are utilized to facilitate electrochemical measurements, all electrodes ( including an Ag/AgCl reference electrode, GCE as the working electrode with a diameter of 2 mm, and a platinum counter electrode ) were selected from Azar Electrode Company (Urmia, Iran). Surface morphology was studied by field emission scanning electron microscopy (FE-SEM) and elemental anlysis was implemented by energy dispersive X-ray analysis (EDX) using TESCAN MIRA3 FEG-SEM (Czech Republic). Specific surface area and pore size distribution determined by Brunauer–Emmett–Teller (BET) and Barrett-Joyner-Halenda (BJH) with BELSORP MINI II, BEL, Japan and the crystalline phase of γ.MnO₂-CS characterized by XRD using PW1730,Philips, Netherlands with CuKα radiation (λ = 1.54056 nm), the XRD pattern was recorded in range of (10–80)°. A Bruker instrument (Billerica, Massachusetts, USA) was utilized to acquire Fourier Transform Infrared (FT-IR) spectra. Dynamic light scattering (DLS)/Zeta potential (Zeta) of the dispersed materials was determined by Nanotrac Wave instrument (Microtrac, Germany). Atomic force microscopy (AFM) analysis of the particles was performed by the Nanosurf AG Gräubernstrasse 124,410 Liestal (Switzerland).

### Methods

All research methods were performed following the relevant guidelines and regulations. Also, this research received approval from the ethics committee at Tabriz University of Medical Sciences.

#### Synthesis approach of γ.MnO2-CS

The γ.MnO_2_-CS composite was prepared using a method described in a previously published study^34^. Breifly, a 60 g/L solution of KMnO_4_ was slowly added to a mixture of 0.3 g CS, 4 mL ethanol, and 2 mL water^35^. The mixture of KMnO_4_/CS was stirred vigorously for at least 8 h at room temperature. Next, the precipitate of γ.MnO_2_-CS was then filtered, washed with distilled water, and then dried at 60 °C for 12 h. Then, 2.5 mg of the dried γ.MnO_2_-CS was dispersed in distilled water (5 mL) and sonicated for about 5–10 min.

#### Synthesis approach of citrate modifided-AuNPs

50 mL of HAuCl_4_ solution (0.5 mM) was boiled and mixed sodium citrate (5 mL of 38.8 mM). The addition of sodium citrate into the gold solution changes the yellow color of the solution into wine red. Next, the mixture was stirred until the color was fixed. The solution was then filtered to remove any solid particles^36,37^. The produced AuNPs concentration was determined by the Beer-Lambert equation.^14,38^.

#### Serum samples collection

Blood samples were collected via venous blood. The samples were allowed to clot for 30 min at room temperature before centrifugation. The samples were centrifuged at 3000 rpm for 10 min to separate the serum. The serum was then aliquoted and stored at − 20 °C until analysis. No preservatives or stabilizers were added. The samples were collected from five smokers (four males and one female) aged 25–84 years and samples were randomly selected to ensure unbiased analysis. All samples are obtained from healthy individuals except sample number 5 which is a heavy smoker and a cancer patient. This project was approved by the Research Ethics Committee of Tabriz University of Medical Sciences ethics committee (license code: IR.TBZMED.REC.1402.580). Informed consent was obtained from all subjects and/or their legal guardian(s).

#### Fabrication of immunosensor

**Fig. 1:**
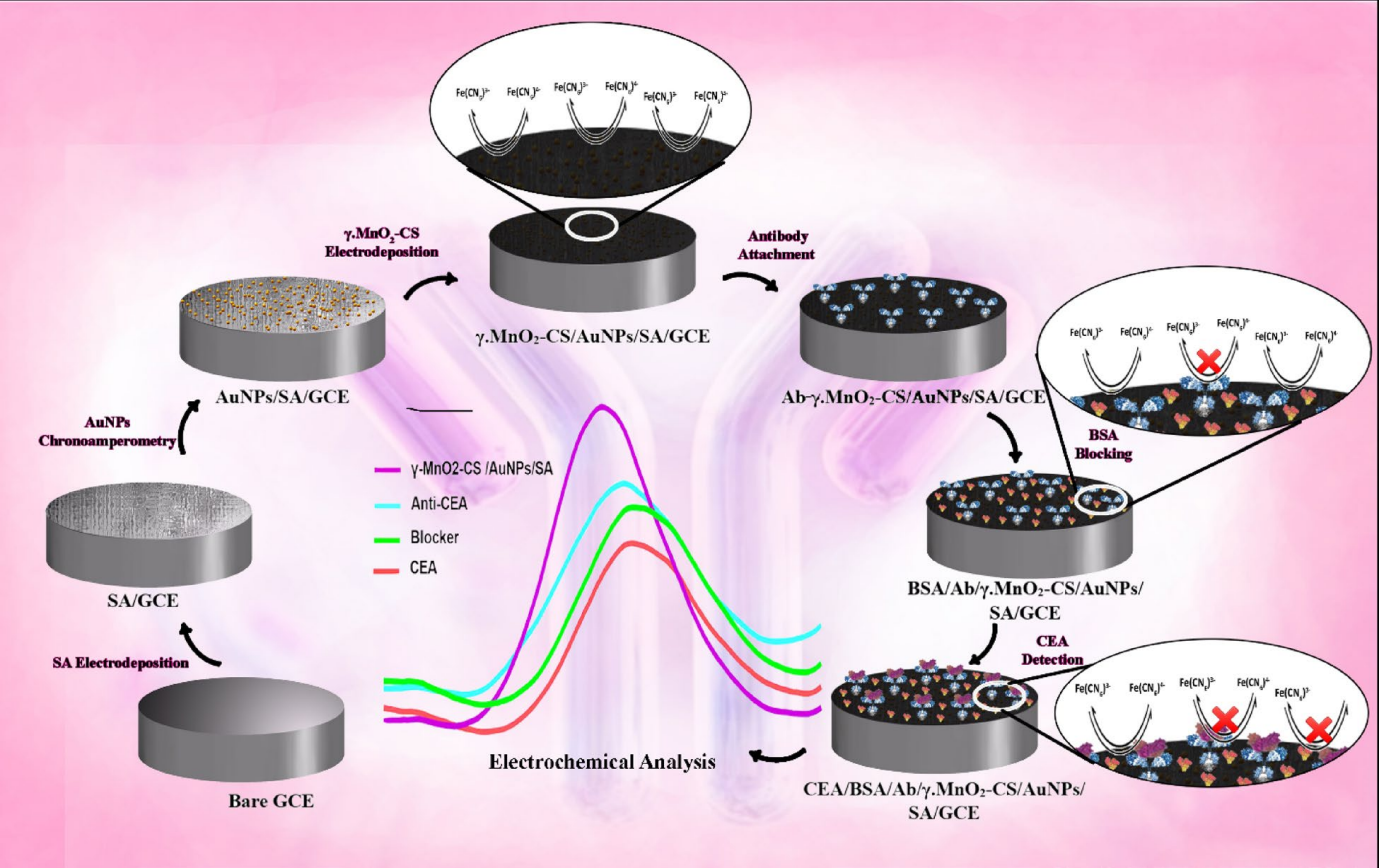
Construction steps. Graphic illustration of the construction steps of the immunosensor.

The electrochemical synthesis of the polymer layer on the surface of the clean GCE was performed as follows (Fig. 1). To begin with, the bare GCE was polished using a soft cloth and completely rinsed with distilled water. Then, as the first step of electrode surface modification, SA was electrodeposited on the surface of bare GCE by CV technique using a potential window from − 1.8 to + 1.8 V for 25 cycles with a sweep rate of 100 mV s^− 1^. In the next step, AuNPs were immobilized on the modified GCE by the chronoamperometric method. Subsequently, γ.MnO_2_-CS was electrodeposited in a potential window of -1.5 to + 1.5 V (10 cycles and 100 mV s^− 1^). 5 µL of Ab (200 µg/mL) was drop coated onto the γ.MnO_2_-CS/AuNPs/SA/GCE surface followed by incubating at 4 °C for 30 min. Then, the electrode washed with PB solution (50 mM, pH 7.5). To block any leftover active sites and decrease non-specific binding, 5 µL of BSA (10 mg/mL) solution was applied to the /Ab/γ.MnO_2_-CS/AuNPs/SA/GCE electrode surface and allowed to incubate at ambient temperature for 1 h^39^. Finally, after washing with PB solution, different CEA concentrations (5 µL) were dropped onto the BSA/Ab/γ.MnO_2_-CS/AuNPs/SA/GCE and incubated at 4 °C for 50 min.

#### Immunoassay approach

CEA at different concentrations was incubated with the fabricated electrode and kept at 4 °C for 50 min to complete the interaction with Ab. To remove any unwanted binding, the CEA/BSA/Ab/γ.MnO_2_-CS/AuNPs/SA/GCE electrode was rinsed with 50 mM PB solution. The electrochemical signals were then analyzed using the DPV technique in [Fe(CN)₆]³⁻/⁴⁻-supported buffer solution. The analytical signals were calculated using the equation: Ratio = I_0_/I_t_, in which I_0_ and I_t_ are the developed probe current in the absence and presence of CEA, respectively.

## Results and discussion

### Physical characterization of the constructed electrodes

The morphology and structure of the nanocomposites were evaluated using various analytical techniques. FE-SEM and AFM provided high-resolution images of the sample surface, revealing the morphology, size, and shape of particles or structures, as well as mechanical properties at the nanoscale. Surface area and pore size distribution were assessed using BET/BJH. To determine the elemental composition and quantify the presence of each element EDX was utilized. DLS/Zeta measurements were used to analyze particle size distribution and surface charge. The functional groups present in the samples were revealed using FT-IR spectroscopy.

#### FE-SEM results

**Fig. 2:**
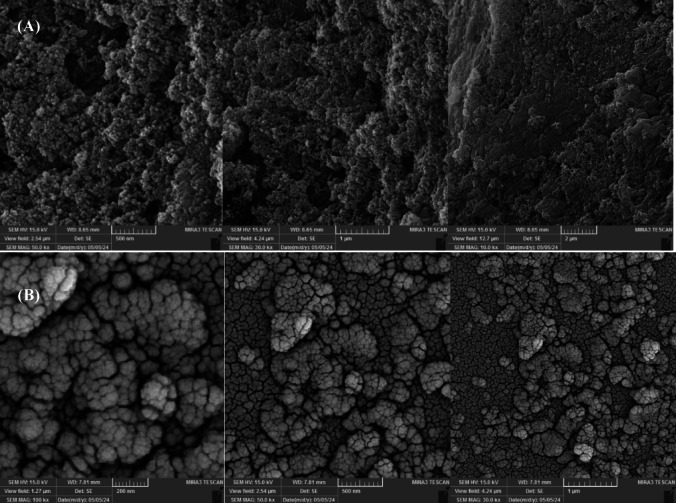
FE-SEM images. FE-SEM images of γ.MnO_2_-CS (**A**) and γ.MnO_2_-CS/AuNPs/SA/GCE (**B**).

The morphology of γ.MnO₂-CS and γ.MnO₂-CS/AuNPs/SA/GCE was investigated using FE-SEM (Fig. 2A,B). The γ.MnO₂-CS nanomaterials exhibited a porous and rough surface, which is advantageous for adsorption. The FE-SEM image of the γ.MnO₂-CS revealed a heterogeneous morphology (Fig. 2A). The surface was characterized by a dense, irregular network of γ.MnO₂ particles embedded within the CS matrix. The γ.MnO₂ particles exhibited a range of sizes and shapes, with some forming agglomerates of smaller particles. These agglomerates were interspersed with individual, more spherical γ.MnO₂ particles. The image indicated that the γ.MnO₂ particles were well-dispersed within the CS matrix, which could contribute to the composite’s surface area and potential for enhanced functionality. The presence of pores and voids, visible as lighter areas in the image, suggested a porous structure. This porosity, likely due to the CS matrix, may play a critical role in applications requiring molecular or ionic diffusion within the material. Overall, the FE-SEM image revealed a highly porous and heterogeneous structure for the γ.MnO₂-CS composite, underscoring its potential for applications requiring a high surface area, enhanced mass transport, or catalytic activity. To further quantify the particle size distribution of γ.MnO_2_-CS observed in the FE-SEM images, a histogram was constructed which reveals a relatively narrow size distribution with an average particle diameter of approximately 25 nm. This quantitative analysis complements the qualitative morphological observations and confirms the uniformity of the particle sizes.

Figure 2B depicts a complex, multilayer structure formed on the GCE through layer-by-layer deposition of SA, AuNPs, and γ.MnO₂-CS. The image shows a rough, porous surface, likely corresponding to the underlying GCE. This rough surface provided a foundation for subsequent layer adhesion. While the SA layer itself was not directly visible, its presence was inferred from the encapsulation of other layers. The overall structure appeared more cohesive and less porous than the underlying GCE, suggesting that the SA layer acted as a binder, creating a stable matrix for the other components. Smaller, brighter, and more densely packed particles were observed on the surface, indicating the presence of AuNPs. These nanoparticles appeared evenly distributed and likely dispersed among the γ.MnO₂-CS particles, suggesting successful integration of AuNPs into the structure. The γ.MnO₂-CS particles were relatively large compared to the underlying GCE and appeared dispersed throughout the surface, indicating successful deposition of this layer. The combination of these components created a highly functionalized surface with potential applications in sensing, catalysis, and energy storage.

#### BET/BJH analysis

The nitrogen adsorption-desorption isotherm at 77 K is shown in Fig. 1S which is used to investigate the pore structure and surface area of γ.MnO₂-CS. The specific surface area (m^2^/g) and distribution of pore volume in γ.MnO₂-CS were determined by BET and BJH-analysis (Table S1). The BET method was employed to measure the sample’s surface area while the BJH method was used to analyze the pore size distribution. the γ.MnO₂-CS composite displayed a surface area of 30.055 m²/g according to the BET method. The total volume of pores, determined at p/p0 = 0.990, equaled 0.1941 cm³/g. The average diameter of the pores, derived from the BET data was estimated to be 25.834 nm. BJH analysis of the adsorption branch of the isotherm indicated a mesoporous structure, with a peak pore radius at 12.22 nm. The total pore volume calculated with the BJH approach was 0.1961 cm³/g, which aligns with the value obtained from the BET analysis. Based on these results and previous studies, it is concluded that γ.MnO₂-CS possesses a mesoporous nature as pore diameters range from 2 to 50 nm^40,41^.

The mesoporous characteristic of the γ.MnO₂-CS composite, with its moderate surface area and pore volume, is expected to enhance the immobilization of antibodies. The increased surface area provides more binding sites for antibody attachment, leading to a higher sensitivity of the immunosensor. Furthermore, the mesoporous structure can reduce steric hindrance, allowing for efficient binding of the analyte to the immobilized antibodies, ultimately improving the sensor’s response.

#### XRD analysis

**Fig. 3:**
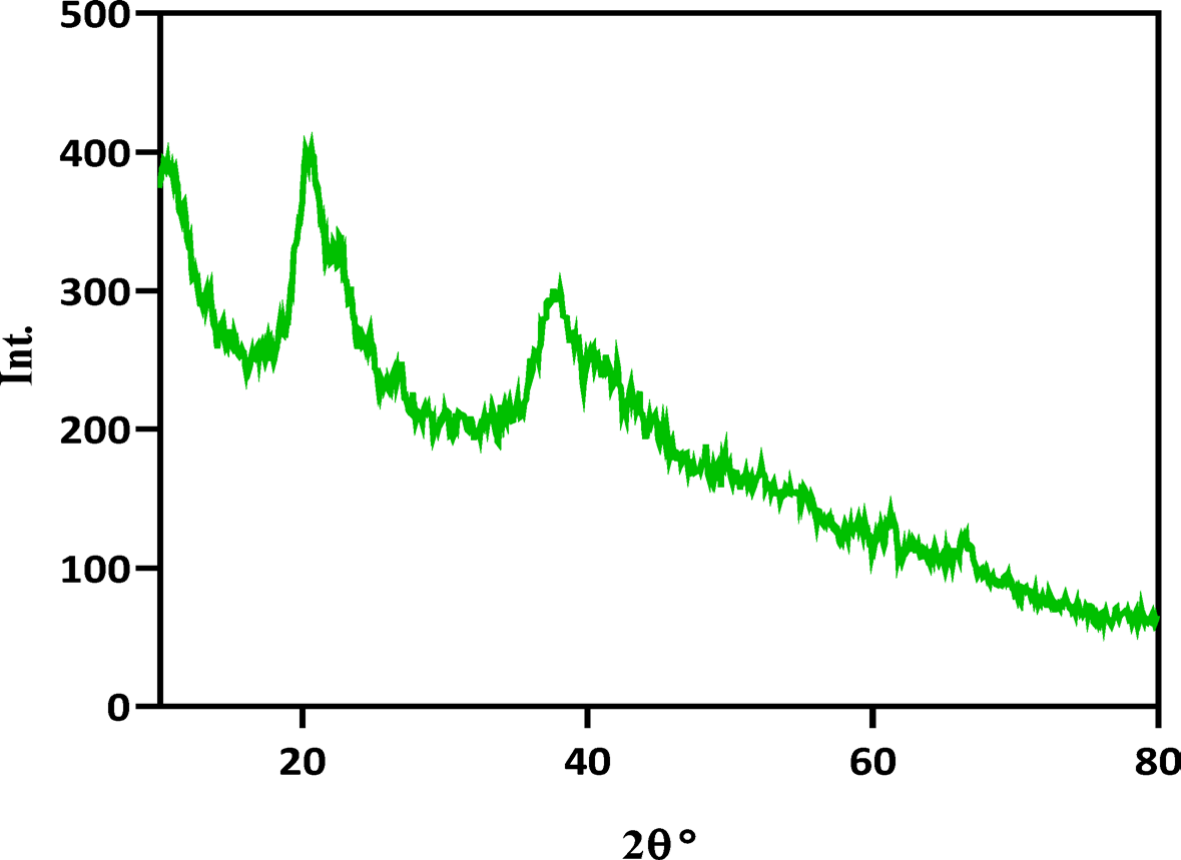
XRD analysis. XRD pattern of γ.MnO_2_-CS.

The XRD pattern of γ.MnO_2_-CS is displayed in Fig. 3. The γ.MnO_2_-CS spectrum displays a distinct characteristic peak at 2θ = 20.13° corresponding to CS. Chitosan shows a peak at 2θ = 10°, but this peak is weak in XRD pattern of γ.MnO_2_-CS^42^. The wide peak indicates that CS in the composit is amorphous, which is consistent with finding from previous studies. A peak is observed in the 35–40° 2θ range, which may indicate the presence of γ-MnO₂. Based on studies γ-MnO₂ is typically shows diffraction peaks at 22°, 57°, and 68.6°, but the low intensity and significant broadening of this feature suggest that the γ-MnO₂ phase possesses very low crystallinity. This is consistent with the understanding that γ-MnO₂ generally has lower crystallinity and small crystallite size compared to other MnO₂ polymorphs such as α-MnO₂ and β-MnO₂. Furthermore, adding chitosan into the composite is expected to further alter the crystalline order of the γ-MnO₂ phase, potentially leading to amorphization. The interaction between chitosan and γ-MnO₂, as evidenced by the observed peak broadening, indicates that chitosan has been successfully incorporated into the material, influencing the overall structure^34,43,44^.

#### EDX analysis

The elemental compositions of γ.MnO₂-CS/AuNPs/SA/GCE and γ.MnO₂-CS were determined using EDX analysis. The spectra are shown in Fig. 3S, and the extracted data are presented in Table 1A,B. The EDX spectrum of γ.MnO₂-CS exhibited peaks at 0.5 keV and 5.9 keV, corresponding to oxygen (O) and manganese (Mn), respectively. Peaks at 0.3 keV and 3.3 keV were attributed to carbon (C) and potassium (K) (Fig. 2S(B)). These results confirmed the successful loading of MnO₂ nanoparticles onto CS^41^.

#### DLS analysis and zeta potential

Figure 3S(A) shows the DLS histogram, indicating that the hydrodynamic diameter of γ.MnO₂-CS is around 75 nm. The particle size observed by FE-SEM was smaller than that obtained by DLS analysis, which can be attributed to the measurement of the hydrodynamic diameter in DLS^45^. As shown in Fig. 3S(B), the zeta potential of the synthesized nanocomposite was − 10.3 mV, indicating a negative surface charge. Moreover, γ.MnO₂-CS exhibited a narrow size distribution (polydispersity index = 0.351) with an average particle size of 75.37 nm. The difference in particle size obtained from FESEM and DLS can be attributed to the fundamental distinctions between the two techniques. FESEM measures particle size in a dry state, whereas DLS assesses particle size in an aqueous medium. Additionally, the high hydrophilicity of CS and its potential to form hydrogels can lead to a significant difference in measurements between these two methods^46^.

#### FT-IR analysis

The FT-IR spectra of γ.MnO₂-CS nanohybrids in the range of 500–3500 cm⁻¹ are shown in Fig. 3S(C). The spectrum displayed characteristic peaks of CS and MnO₂. Peaks at 3751.20 cm⁻¹ and 3388.08 cm⁻¹ were attributed to the O-H stretching vibrations of hydroxyl functional groups in CS and MnO₂, respectively. The peak at 2924.79 cm⁻¹ was assigned to the C-H stretching vibrations of alkyl groups in CS. The peak at 1623.29 cm⁻¹ represented C = O stretching vibrations of the amide group in CS. Peaks at 1411.14 cm⁻¹ and 1062.59 cm⁻¹ corresponded to C-N and C-O stretching vibrations in the pyranose ring of CS, respectively. The presence of these characteristic peaks confirmed the successful synthesis of the γ.MnO₂-CS composite^34,47,48^.

#### AFM results

**Fig. 4:**
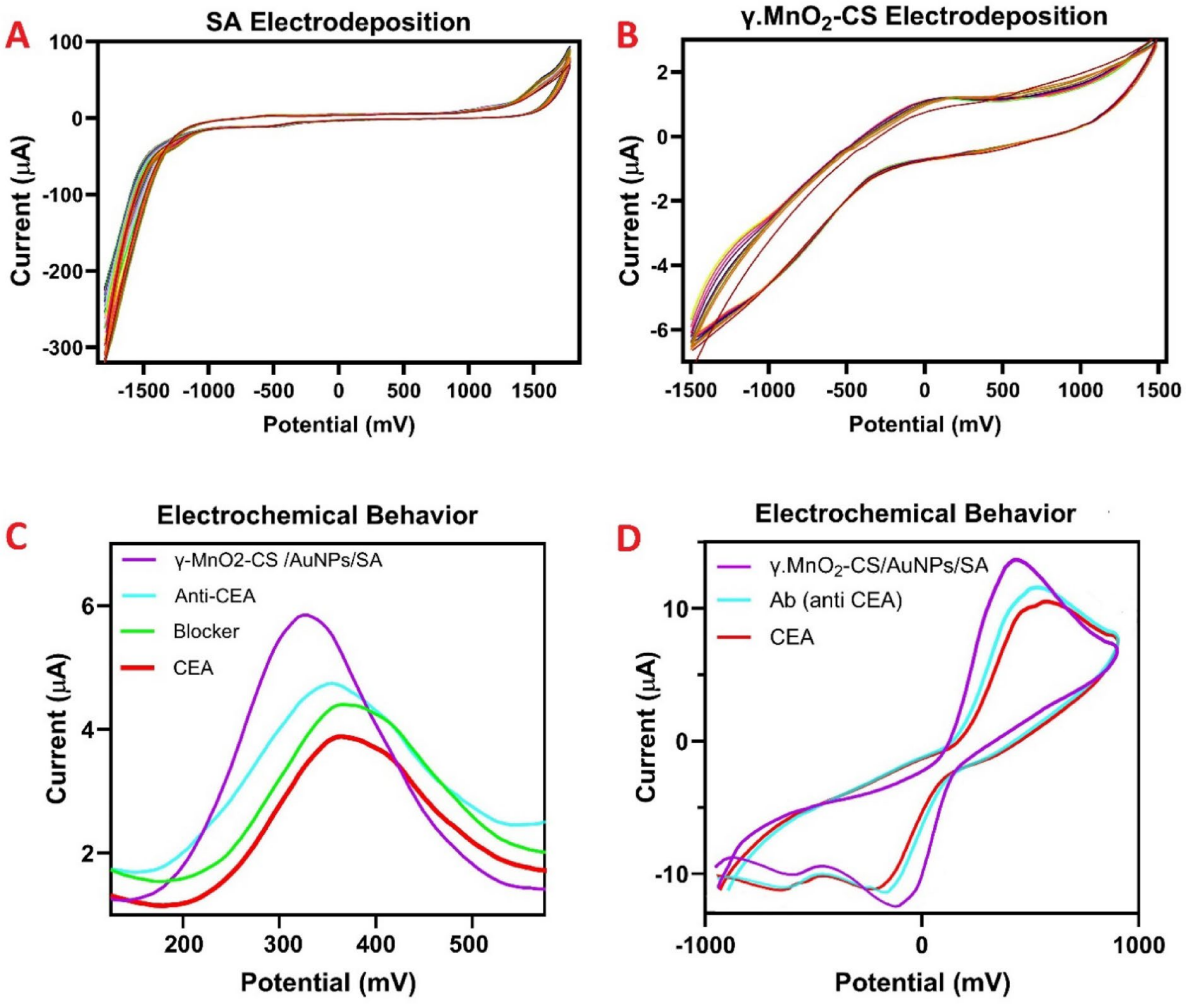
Electrodeposition curves (ECs). ECs of SA (**A**) and γ.MnO_2_-CS (**B**). DPV voltammograms of γ.MnO_2_-CS/AuNPs/SA/GCE, Ab/γ.MnO_2_-CS/AuNPs/SA/GCE, BSA/Ab/γ.MnO_2_-CS/AuNPs/SA/GCE, and CEA/BSA/Ab/γ.MnO_2_-CS/AuNPs/SA/GCE (**C**). CV voltammograms of γ.MnO_2_-CS/AuNPs/SA/GCE, Ab/γ.MnO_2_-CS/AuNPs/SA/GCE, and CEA/BSA/Ab/γ.MnO_2_-CS/AuNPs/SA/GCE in [Fe (CN)_6_]^3–/4–^ as a redox probe with pH 7.5 (**D**). Ab and CEA concentrations were 200 µg/mL and 1 ng/mL respectively.

The surface roughness patterns, morphology, and topography of the γ.MnO₂-CS nanocomposite were analyzed using the AFM method. The image reveals a heterogeneous surface with distinct peaks and valleys, indicating the presence of γ.MnO_2_ nanoparticles embedded within the chitosan matrix. The peaks (bright regions) correspond to the γ.MnO_2_ nanoparticles, while the valleys (dark regions) represent the chitosan matrix. The γ.MnO_2_ nanoparticles appear to be well-dispersed within the chitosan matrix, with minimal agglomeration. The uniform distribution of nanoparticles suggests effective synthesis and integration of the γ.MnO_2_ particles into the chitosan matrix. This image shows a topography scan with a polynomial fit applied, which highlights a surface roughness. The topography profile shows the height variation across the scanned surface, indicating surface roughness. The y-axis displays the measured height (in nm), while the x-axis represents the distance across the scanned area (in µm). Notably, there’s a slight upward slope indicated in the data, which could suggest a variation in the distribution of the nanocomposite (Fig. 4S(A,B)). Figure 4S(C) representation provides a more detailed view of the surface morphology, showcasing peaks and valleys, which are characteristic of nanostructured materials. The shading and color gradients represent height variations and may highlight where the γ.MnO_2_ particles are distributed relative to the chitosan matrix.

### Electrochemical investigations and mechanism of detection

Figure 4A,B show the CV curves for the electrodeposition of SA and γ.MnO₂-CS, respectively. After sensor preparation, Ab, BSA, and CEA were sequentially immobilized on the sensor. DPV was chosen as a robust method to investigate the electrochemical properties of the prepared electrodes at each modification step. Figure 4C shows the DPV curves of the γ.MnO₂-CS/AuNPs/SA/GCE electrode and bare GCE in a 50 mM [Fe(CN)₆]^³⁻/⁴⁻^ solution, within a potential window from − 1.0 to + 1.0 V with a sweep rate of 100 mV s⁻¹. The DPV results indicated that the electrochemical response was significantly enhanced by the deposition of nanocomposites. Figure 4D shows the CV curves for the same sensor preparation steps. By electrodepositing SA onto a bare GCE, a thin layer is formed that serves as an initial scaffold for further optimization of the electrode surface. The subsequent deposition of gold not only enhances the active surface area of the electrode but also improves its electrocatalytic properties. Additionally, the introduction of γ.MnO₂-CS increases the specific surface area of the electrode and provides a primary substrate for the binding od PSA antibody. The functional groups and structure of MnO_2_-CS in the nanocomposite facilitate the binding of antibodies to the optimized electrode surface. As illustrated in Fig. 1, when an antigen binds to the antibody on the surface of BSA/Ab/γ.MnO₂-CS/AuNPs/SA/GCE and forms an immunocomplex on the electrode surface, the oxidation current of the [Fe(CN)₆]^³⁻/⁴⁻^ decreases. This decrease is attributed to the binding affinity between CEA and the immobilized Ab on the BSA/Ab/γ.MnO₂-CS/AuNPs/SA/GCE electrode, which hindered electron transfer at the electrode surface, resulting in a decrease in probe current. The current ratio was calculated by dividing the oxidation peak current before binding (I_Ab_) by the oxidation peak current after binding (I_Ab−Ag_)^49^.

### Optimization

**Fig. 5:**
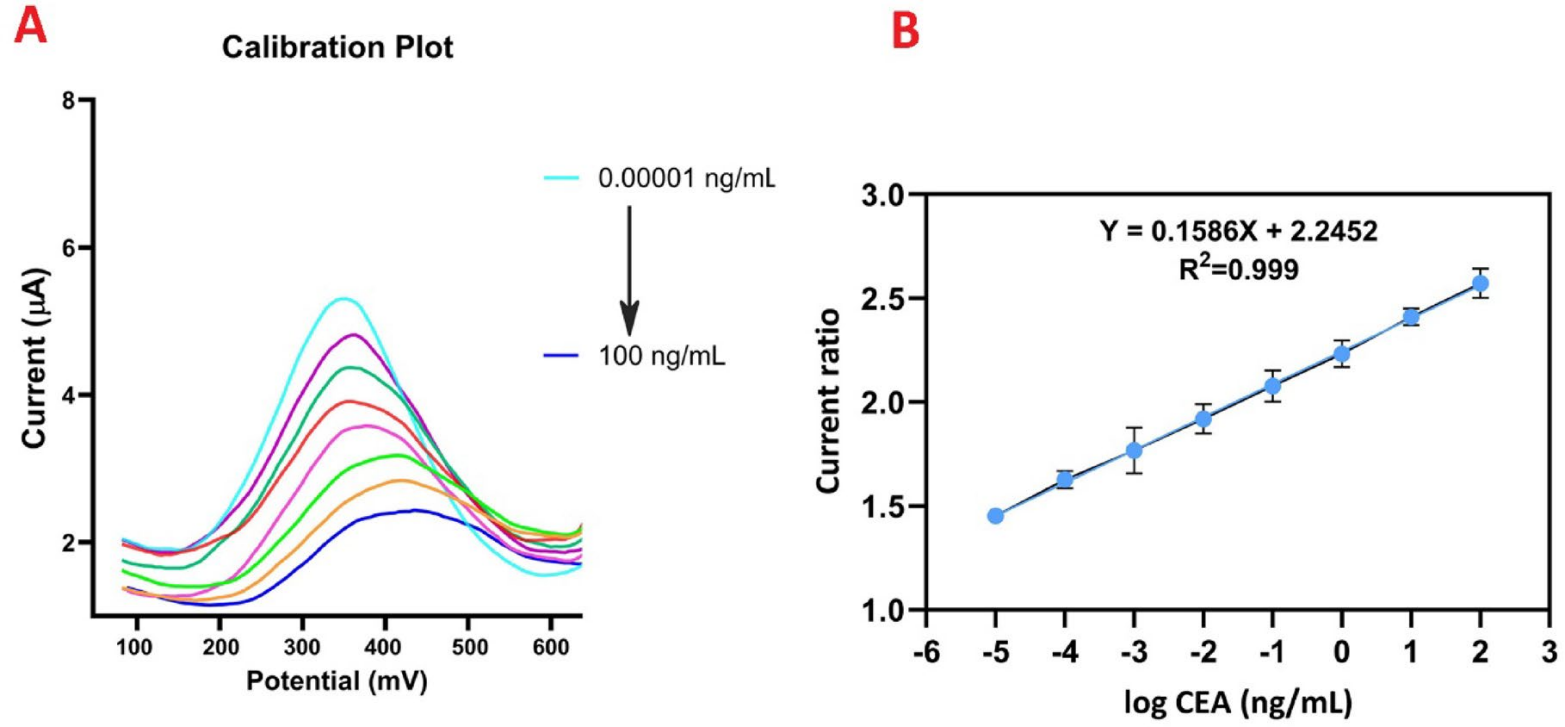
Calibration. DPV responses of the immunosensor to different concentrations of CEA from top to down: 0.00001, 0.0001, 0.001, 0.01, 0.1, 1, 10, 100 ng/mL (**A**). The calibration curve in different concentrations of CEA (**B**).

To achieve optimal analytical performance of the fabricated immunosensor, key experimental parameters such as the concentration of SA and γ.MnO₂-CS, the number of electrodeposition cycles, and the chronoamperometric potential of AuNPs were investigated (Fig. 5S). Optimal conditions were selected based on the maximum gain of the nanocomposite. Different concentrations of SA (1.25, 2.5, and 5 mM) (Fig. 5S(A)) and γ.MnO₂-CS (250, 500, and 1000 µg/mL) (Fig. 5S(B)) were prepared and used to modify the GCE. The optimal concentrations were determined to be 2.5 mM for SA and 500 µg/mL for γ.MnO₂-CS. The effect of varying the number of CV cycles during electrodeposition was also studied, with 25 cycles for SA and 10 cycles for γ.MnO₂-CS identified as optimal (Fig. 5S(C,D). Similarly, for the electrodeposition of AuNPs by chronoamperometry, the potential (-500, 0, and + 500 mV) was optimized, with 0 mV selected as the optimal potential (Fig. 5S(E)).

The bioactivity of CEA and Ab is strongly influenced by pH. The function of the immunosensor was analyzed at various pH values of [Fe(CN)₆]³⁻/⁴⁻ at ambient temperature. The current ratio increased as the pH increased from 5.5 to 7.5 and then decreased significantly from 7.5 to 9.5 (Fig. 5S(F)). The optimal current change was observed at pH 7.5, as acidic or alkaline conditions can disrupt the Ab-Ag bond and degrade protein materials^50^. Therefore, pH 7.5 was used for subsequent experiments.

The sensitivity of the BSA/Ab/γ.MnO₂-CS/AuNPs/SA/GCE-based immunosensor was strongly influenced by the duration of Ab and CEA incubation. To determine the optimum incubation time for Ab immobilization, signal measurements were performed at intervals ranging from 10 to 60 min (Fig. 5S(G)). BSA was used as a blocking agent to decrease non-specific interactions, with an optimal blocking time of 60 min identified (Fig. 5S(H)). The incubation time between the modified electrode and CEA (1.0 ng/mL) was also investigated over a range of 10 to 60 min (Fig. 5S(I)). The current increased with incubation time and then stabilized, indicating balanced binding between Ab and CEA. Based on these results, 50 min was determined to be the best incubation time for CEA-Ab interaction.

### Analytical performance and sensitivity

The analytical performance of the γ.MnO₂-CS/AuNPs/SA functionalized probe was evaluated using DPV technique. Figure 5A shows the DPV peak currents for different CEA concentrations under the optimum conditions. As shown, the current decreased with increasing CEA concentration due to the interaction between Ab and CEA on the surface of the probe, which impeded electron transfer between the electrode and electrolyte^51^. As shown in Fig. 5B, the current ratio correlated with the log of CEA (log CEA) concentration over a range of 10 fg/mL to 0.1 µg/mL, following the equation: current ratio (µA) = 0.1586 log CEA + 2.245. The calculated limit of detection (LOD) and limit of quantitation (LOQ) of the probe were as 9.57 fg/mL and 31.6 fg/mL, respectively.

A comparison of various CEA detection techniques is presented in Table 2A. Techniques such as chemiluminescence immunoassays, fluorometric analysis, and photoelectrochemical immunoassays have been used for CEA detection in dfferent media^52^. However, these methods often require multiple detection steps, time-consuming procedures, and labeling, which can interfere with target binding and lead to inaccurate results. In contrast, label-free immunosensors, such as the one developed in this study, offer advantages in terms of simplicity and sensitivity. Voltammetric techniques, particularly DPV, provide higher sensitivity and selectivity, enabling the detection of lower CEA concentrations with greater accuracy^17,53^. While upconversion nanoparticles (UCNPs) have been used in luminescence-based methods due to their advantages, their synthesis is complex and environmentally risky due to the potential release of toxic heavy metals. In contrast, the synthesized nanoprobes in this study offer simplicity, cost-effectiveness, and environmental safety^49^.

### Repeatability, reproducibility, and stability

The repeatability and accuracy of the electrochemical immunosensor were evaluated by measuring three concentrations of CEA (1, 0.01, and 0.0001 ng/mL). The relative error percentage (RE%) and relative standard deviation (RSD) were − 11.51% and 6.76%, respectively, demonstrating good repeatability and accuracy (Table 2B). Reproducibility was assessed using four independently modified electrodes, with RSD values of 2.6%, 2.95%, and 4.94% for the tested concentrations, indicating efficient reproducibility. The stability of the Ab/γ.MnO₂-CS/AuNPs/SA-modified GCE was evaluated by monitoring the CV peak current over multiple cycles. The peak current remained stable, indicating consistent performance (Fig. 6S(A)). Long-term stability was assessed by storing the fabricated immunosensor at 4 °C for 7 days. The peak current showed minimal changes (1.04% after 3 days and 2.08% after 7 days), confirming the immunosensor’s stability for CEA detection (Fig. 6S(B)).

### Selectivity and interference study

The selectivity of the Ab/γ.MnO₂-CS/AuNPs/SA/GCE-based immunosensor for CEA (1.0 ng/mL) was evaluated using potential interfering biomolecules, including vitamin C, glucose, glycine, and tryptophan. The relative error was calculated by comparing DPV signals in the presence and absence of these biomolecules (Table 1C). No significant interference was observed due to their low anodic and cathodic potentials. The selectivity and specificity of the immunosensor were further confirmed using BSA and prostate-specific antigen (PSA). The minimal deviation in response underscored the immunosensor’s excellent selectivity, attributed to the high binding affinity between Ab and Ag (Fig. 6S(C)).

### Application of the developed probe

#### Spiked samples

The practical applicability of the Ab/γ.MnO₂-CS/AuNPs/SA/GCE immunosensor was assessed by measuring CEA levels in the serum matrix. The serum was centrifuged and diluted 10-fold with PB solution (pH 7.4). As in the calibration step, known concentrations of antigen were prepared and dissolved in the diluted serum. Then, the received DPV signal was measured using the designed sensor. Based on the test results, RSD was calculated. The RSD below 5% indicates the ability of the biosensor to reliably measure the specific analyte in complex matrices. Calculation of recovery values in serum sample analysis using an immunosensor includes comparing the measured concentration of the analyte in a sample with a known concentration. Percentage recovery is a measure of how much of the analyte was successfully detected by the sensor. The initial concentration of CEA in the serum sample before adding the specified concentrations of CEA was either zero or very negligible. Therefore, the recovery percentage was calculated using the following formula: Recovery (%)= (calculated CEA- spiked CEA) /spiked CEA ×100. The obtained results showed that the RSD% values varied between 2.17 and 4.11%, and the recovery values ranged from 95.47 to 103.25% (Table 3). These findings showed the practicality of the proposed Ab/γ.MnO₂-CS/AuNPs/SA/GCE immunosensor as a powerful tool for early detection. .

**Table 1:** EDX analyses and interferer study.

(A) EDX analyses of γ.MnO_2_-CS/AuNPs/SA/GCE			
Element		Weight%	Atomic %
C		86.98	92.39
N		4.92	4.48
O		3.54	2.82
Mn		0.10	0.02
Au		4.47	0.29
		100.00	100.00

(B) EDX analyses of γ.MnO_2_-CS			
Element		Weight%	Atomic %
C		24.21	40.01
N		6.87	9.73
O		28.03	34.77
K		4.92	2.50
Mn		35.97	12.99
		100.00	100.00

(C) Interferer effect on the detection signal.			
Interferer	Amount	Signal changes percentage	
Vitamin C	15 ng/mL	-13.86%	
Glucose	1.5 mg/mL	-16.13%	
Glycine	45 ng/mL	-15.91%	
Tryptophan	30 ng/mL	15.86%	

**Table 2:** Comparison of the proposed immunosensors and the accuracy of the developed method.

A) Comparison of the proposed immunosensors with other reported electrochemical methods for CEA detection.							
Electrode	Detection technique		Linear range		LOD		Ref.
Nano-Au/Chit/NG/GCE Au/PDCNTs/(PSS/ PDCNTs)_2_ /GCE	Amperometric Amperometric		0.2 to 120 ng/mL 0.1 to 2.0 ng/mL and 2.0 to 160 ng/mL		0.06 ng/mL		^54^
					0.06 ng/ml		^55^
GR-IL/pPt/GCE	ECL		0.001 fg/mL to 1 ng/mL		0.0003 fg/mL		^56^
Au@Ce_2_Sn_2_ O_7_ /GCE	ECL		0.001 to 70 ng/mL		0.53 fg/mL		^57^
Flower-like Au@BSA	ECL		0.001 to 200 ng/mL		0.0003 ng/mL		^58^
AuNPs/PDDA/rGO-BaYF5:Yb, Er/GCE	ECL		0.001 to 80 ng/mL		0.87 pg/mL		^59^
3DPt/HGO/GCE	DPV		0.001to150 ng/mL		0.0006 ng/mL		^60^
Cu_2_S/Pd/CuO/GCE	CV and EIS		0.0001 ng/mL to 100 ng/mL		33.11 fg/mL		^61^
Graphene nanocomposites/AuNPs/GCE	CV and EIS		0.001to150 ng/mL		0.0006 ng/mL		^62^
PtPd/N-GQDs@Au/GCE	CV and EIS		5 fg/mL to 50 ng/mL		2 fg/mL		^63^
rGO/GCE	CV and EIS		0.1 to 5 ng/mL		0.05 ng/mL		^64^
AuNPs/p(Py-co-EDOT) /PGE	DPV		0.001 to 100 ng/mL		0.741 pg/mL		^65^
AuNPs/THi/rGO/GCE	CV and DPV		10 to500 pg/mL		4 pg/mL		^66^
AuNPs/PEDOT/GR/GCE	CV, EIS and DPV		0.0004 to 40 ng/mL		0.1 pg/mL		^67^
(PB-AuNPs/rGO-MWCNTs)n/GCE	DPV		0.2 to 1.0, and 1.0 to 40.0 ng/mL		60 pg/mL		^68^
AuNPs/NB-ErGO/GCE	CV and DPV		0.001 to 40 ng/mL		0.00045 ng/mL		^69^
γ.MnO_2_-CS /AuNPs/SA/GCE	CV and DPV		0.00001 to 100 ng/mL		9.57 fg/mL		This study

(B) Accuracy and precision of the developed γ.MnO_2_-CS/AuNPs/SA method for CEA detection							
Nominal concentration (ng/mL)	Precision (RSD%)			Accuracy (RE%)			
	Intraday	Interday		Intraday		Intraday	
1	12.0	2.9		-10.0		-6.6	
0.01	13.8	2.8		-14.6		-10.7	
0.0001	4.3	4.6		-13.8		13.4	

Interday precision (RSD%), Intraday accuracy (RE%), NG: nano-structure gold; ECL: electrochemiluminescence; GR-IL: ionic liquid functionalized graphene film; pPt: porous platinum; PDDA: poly (diallyldimethylammonium chloride); 3DPt: three dimensional porous graphene oxide supported platinium metal nanoparticles; N-GQDs : nitrogen-doped graphene quantum dots; p(Py-co-EDOT):3,4-ethylenedioxythiophene (EDOT), and pyrrole (Py); THi: Thionin; PEDOT: Poly (3,4-ethylenedioxythiophene); GR: Graphene; PB: Prussian blue; MWCNTs: Multi-walled carbon nanotubes with carboxylic acid groups; NB: Nile blue; ErGO: Electrochemically reduced graphene oxide.

**Table 3:** Detection of CEA in serum sample using standard addition method.

Sample	Spiked (ng/mL)	Founded (ng/mL)	RSD (%)	Recovery (%)
Diluted serum	0.0001	0.0001	4.11	97.87
	0.0010	0.00099	2.77	99.27
	0.0100	0.0101	3.91	100.99
	0.1000	0.1030	3.26	103.00
	1.0	1.0325	2.83	103.25
	10.0	9.5470	2.17	95.47

#### Pateint samples

The immunosensor’s performance was evaluated by analyzing serum samples from four men and one woman, all smokers (ages 65, 32, 63, 25, and 84). All the samples were collected from healthy individuals, except for sample number 5, who is both a heavy smoker and a cancer patient. As detailed in Sect. 2.3.4, after the sensor was constructed, 5 µL of these serum samples were drop-casted, and then the amount of CEA was determined using the formula provided in Sect. 3.4. The findings were compared to those obtained using the ELISA reference method. According to Table 4, the results from the immunosensor were consistent with ELISA, confirming its effectiveness for CEA detection in real blood samples.

**Table 4:** Comparison of CEA measurement accuracy of manufactured immunosensor with ELISA method in real samples.

Sample	Age	Gender	Measured CEA concentration using immunosensor(ng/mL)	Measured CEA concentration using ELISA (ng/mL)	T-test
1	65	Male	1.27	1.25	0.78
2	32	Male	0.86	0.86	
3	63	Male	1.84	1.80	
4	25	Female	0.82	0.78	
5	84	Male	6.36	5.22	

## Conclusion

This study presents the development of a label-free electrochemical immunosensor using a γ.MnO₂-CS/AuNPs/SA platform for the sensitive and rapid detection of CEA, a critical biomarker for early cancer diagnosis. High sensitivity and selectivity were made possible by the special nanocomposite structure, which also improved electron transfer and enabled effective antibody immobilization. The immunosensor demonstrated excellent performance in real serum samples, which shows results comparable to the ELISA reference method, highlighting its potential for clinical applications.

Compared to existing methods, this immunosensor offers superior sensitivity, rapid response, and the ability to detect CEA at low levels, making it a potentially useful tool for early cancer diagnosis and timely treatment. The study establishes a novel approach for designing highly selective and sensitive immunosensing devices, paving the way for the detection of other clinically significant biomarkers.

## Electronic supplementary material

Below is the link to the electronic supplementary material.

Supplementary Material 1

## Data Availability

Data will be available upon request. Sajedeh Sobhanparast is responsible for this information.

## References

[1] Zebhauser, R. et al. Identification of a novel group of evolutionarily conserved members within the rapidly diverging murine Cea family. *Genomics* **86** (5), 566–580. 10.1016/j.ygeno.2005.07.008 (2005). 16139472 10.1016/j.ygeno.2005.07.008

[2] Gan, N., Jia, L. & Zheng, L. A sandwich electrochemical immunosensor using magnetic DNA nanoprobes for carcinoembryonic antigen. *Int. J. Mol. Sci.* **12** (11), 7410–7423. 10.3390/ijms12117410 (2011). 22174606 10.3390/ijms12117410 PMC3233412

[3] Beauchemin, N. & Arabzadeh, A. Carcinoembryonic antigen-related cell adhesion molecules (CEACAMs) in cancer progression and metastasis. *Cancer Metastasis Rev.* **32** (3–4), 643–671. 10.1007/s10555-013-9444-6 (2013). 23903773 10.1007/s10555-013-9444-6

[4] Xiang, W., Lv, Q., Shi, H., Xie, B. & Gao, L. Aptamer-based biosensor for detecting carcinoembryonic antigen. *Talanta* **214**10.1016/j.talanta.2020.120716 (2020). 10.1016/j.talanta.2020.120716 32278406

[5] Mahmoudi, T. et al. On-site detection of carcinoembryonic antigen in human serum. *Biosensors* **11** (10). 10.3390/bios11100392 (2021). 10.3390/bios11100392 PMC8534016 34677348

[6] Msagati, T. A. M. Food forensics and toxicology. *Food For. Toxicol.* **2016** 1–436. 10.1002/9781119101406

[7] Hariri, M. et al. Biosensor-Based nanodiagnosis of carcinoembryonic antigen (CEA): an approach to classification and precise detection of Cancer biomarker. *Bionanoscience* **14** (1), 429–446. 10.1007/s12668-023-01250-7 (2024).

[8] Cinquanta, L., Fontana, D. E. & Bizzaro, N. Chemiluminescent immunoassay technology: what does it change in autoantibody detection? *Autoimmun. Highlights* **8** (1). 10.1007/s13317-017-0097-2 (2017). 10.1007/s13317-017-0097-2 PMC5483212 28647912

[9] Goldsmith, S. J. & Radioimmunoassay Review of basic principles. *Semin Nucl. Med.* **5** (2), 125–152. 10.1016/S0001-2998(75)80028-6 (1975). 164695 10.1016/s0001-2998(75)80028-6

[10] Franier, B. D. & La, Thompson, M. Early stage detection and screening of ovarian cancer: A research opportunity and significant challenge for biosensor technology. *Biosens. Bioelectron.* **135**, 71–81. 10.1016/j.bios.2019.03.041 (2019). 31003031 10.1016/j.bios.2019.03.041

[11] Banerjee, P. & Bhunia, A. K. Mammalian cell-based biosensors for pathogens and toxins. *Trends Biotechnol.* **27** (3), 179–188. 10.1016/j.tibtech.2008.11.006 (2009). 19187988 10.1016/j.tibtech.2008.11.006

[12] Mi, F. et al. Application of lectin-based biosensor technology in the detection of foodborne pathogenic bacteria: A review. *Analyst* **146** (2), 429–443. 10.1039/d0an01459a (2021). 33231246 10.1039/d0an01459a

[13] Zhang, L., Guo, W. & Lu, Y. Advances in cell-free biosensors: principle, mechanism, and applications. *Biotechnol. J.* **15** (9). 10.1002/biot.202000187 (2020). 10.1002/biot.202000187 32667120

[14] Soleymani, J., Hasanzadeh, M., Somi, M. H. & Jouyban, A. Differentiation and targeting of HT 29 cancer cells based on folate bioreceptor using cysteamine functionalized gold nano-leaf. *Mater. Sci. Eng. C*. **107**, 110320. 10.1016/j.msec.2019.110320 (2020). 10.1016/j.msec.2019.110320 31761196

[15] Soleymani, J., Hasanzadeh, M., Somi, M. H., Shadjou, N. & Jouyban, A. Highly sensitive and specific cytosensing of HT 29 colorectal cancer cells using folic acid functionalized-KCC-1 nanoparticles. *Biosens. Bioelectron.* **132**, 122–131. 10.1016/j.bios.2019.02.052 (2019). 30870638 10.1016/j.bios.2019.02.052

[16] Cavalcanti, A., Shirinzadeh, B., Zhang, M. & Kretly, L. C. Nanorobot hardware architecture for medical defense. *Sensors* **8** (5), 2932–2958. 10.3390/s8052932 (2008). 27879858 10.3390/s8052932 PMC3675524

[17] Mollarasouli, F., Kurbanoglu, S. & Ozkan, S. A. The role of electrochemical immunosensors in clinical analysis. *Biosensors* **9** (3). 10.3390/bios9030086 (2019). 10.3390/bios9030086 PMC6784381 31324020

[18] Valenti, G. et al. An electrochemiluminescence-supramolecular approach to sarcosine detection for early diagnosis of prostate cancer. *Faraday Discuss.* **185**, 299–309. 10.1039/c5fd00096c (2015). 26394608 10.1039/c5fd00096c

[19] Grieshaber, D., MacKenzie, R., Vörös, J. & Reimhult, E. Electrochemical biosensors - Sensor principles and architectures. *Sensors* **8** (3), 1400–1458. 10.3390/s8031400 (2008). 27879772 10.3390/s80314000 PMC3663003

[20] Cho, I. H., Kim, D. H. & Park, S. Electrochemical biosensors: perspective on functional nanomaterials for on-site analysis. *Biomater. Res.* **24** (1). 10.1186/s40824-019-0181-y (2020). 10.1186/s40824-019-0181-y PMC7001310 32042441

[21] Abbasi, M. et al. An ultrasensitive and preprocessing-free electrochemical platform for the detection of doxorubicin based on tryptophan/polyethylene glycol-cobalt ferrite nanoparticles modified electrodes. *Microchem. J.* **183**10.1016/j.microc.2022.108055 (2022).

[22] Ehsani, M. et al. Low potential detection of doxorubicin using a sensitive electrochemical sensor based on glassy carbon electrode modified with silver nanoparticles-supported poly(chitosan): A new platform in pharmaceutical analysis. *Microchem J.* **165**, 106101. 10.1016/j.microc.2021.106101 (2021).

[23] Hasanzadeh, M. et al. Probing the antigen-antibody interaction towards ultrasensitive recognition of cancer biomarker in adenocarcinoma cell lysates using layer-by-layer assembled silver nano-cubics with porous structure on cysteamine caped GQDs. *Microchem J.* **143**, 379–393. 10.1016/j.microc.2018.08.028 (2018).

[24] Curulli, A. Nanomaterials in electrochemical sensing area: applications and challenges in food analysis. *Molecules* **25** (23). 10.3390/molecules25235759 (2020). 10.3390/molecules25235759 PMC7730649 33297366

[25] Zhu, C., Yang, G., Li, H., Du, D. & Lin, Y. Electrochemical sensors and biosensors based on nanomaterials and nanostructures. *Anal. Chem.* **87** (1), 230–249. 10.1021/ac5039863 (2015). 25354297 10.1021/ac5039863 PMC4287168

[26] Sellimi, S. et al. Structural, physicochemical and antioxidant properties of sodium alginate isolated from a Tunisian brown seaweed. *Int. J. Biol. Macromol.* **72**, 1358–1367. 10.1016/j.ijbiomac.2014.10.016 (2015). 25453289 10.1016/j.ijbiomac.2014.10.016

[27] Sachan, N., Pushkar, S., Jha, A. & Bhattcharya, A. Sodium alginate: the Wonder polymer for controlled drug delivery. *J. Pharm. Res.* **2** (7), 1191–1199 (2009).

[28] Siciliano, G. et al. Beyond traditional biosensors: recent advances in gold nanoparticles modified electrodes for biosensing applications. *Talanta* **268**10.1016/j.talanta.2023.125280 (2024). 10.1016/j.talanta.2023.125280 37862755

[29] Yadav, M., Kaushik, B., Rao, G. K., Srivastava, C. M. & Vaya, D. Advances and challenges in the use of chitosan and its derivatives in biomedical fields: A review. *Carbohydr. Polym. Technol. Appl.* **5**10.1016/j.carpta.2023.100323 (2023).

[30] Rahimi, M. et al. Chitosan-based biomaterials for the treatment of bone disorders. *Int. J. Biol. Macromol.* **215**, 346–367. 10.1016/j.ijbiomac.2022.06.079 (2022). 35718150 10.1016/j.ijbiomac.2022.06.079

[31] Brasselet, C. et al. Modification of chitosan for the generation of functional derivatives. *Appl. Sci.* **9** (7). 10.3390/app9071321 (2019).

[32] Peng, L. et al. Ultrathin two-dimensional MnO2/graphene hybrid nanostructures for high-performance, flexible planar supercapacitors. *Nano Lett.* **13** (5), 2151–2157. 10.1021/nl400600x (2013). 23590256 10.1021/nl400600x

[33] Wang, J. G., Kang, F. & Wei, B. Engineering of MnO2-based nanocomposites for high-performance supercapacitors. *Prog Mater. Sci.* **74**, 51–124. 10.1016/j.pmatsci.2015.04.003 (2015).

[34] Panahandeh, A., Parvareh, A. & Moraveji, M. K. Synthesis and characterization of γ-MnO2/chitosan/Fe3O4 cross-linked with EDTA and the study of its efficiency for the elimination of zinc(II) and lead(II) from wastewater. *Environ. Sci. Pollut Res.* **28** (8), 9235–9254. 10.1007/s11356-020-11359-x (2021). 10.1007/s11356-020-11359-x 33140305

[35] Dinh, V. P. et al. Insight into adsorption mechanism of lead(II) from aqueous solution by Chitosan loaded MnO2 nanoparticles. *Mater. Chem. Phys.* **207**, 294–302. 10.1016/j.matchemphys.2017.12.071 (2018).

[36] Lee, P. C. & Meisel, D. Adsorption and surface-enhanced Raman of dyes on silver and gold sols. *J. Phys. Chem.* **86** (17), 3391–3395. 10.1021/j100214a025 (1982).

[37] Mura, S. et al. Functionalized gold nanoparticles for the detection of nitrates in water. *Int. J. Environ. Sci. Technol.* **12** (3), 1021–1028. 10.1007/s13762-013-0494-7 (2015).

[38] Maye, M. M. et al. Gold and alloy nanoparticles in solution and thin film assembly: spectrophotometric determination of molar absorptivity. *Anal. Chim. Acta*. **496** (1–2), 17–27. 10.1016/S0003-2670(03)00986-3 (2003).

[39] Wang, J. et al. Performances and mechanisms of mg/al and ca/al layered double hydroxides for graphene oxide removal from aqueous solution. *Chem. Eng. J.* **297**, 106–115. 10.1016/j.cej.2016.04.012 (2016).

[40] Tsai, W. C. et al. Removal of copper, nickel, lead, and zinc using chitosan-coated montmorillonite beads in single- and multi-metal system. *Desalin. Water Treat.* **57** (21), 9799–9812. 10.1080/19443994.2015.1035676 (2016).

[41] Dinh, V. P. et al. Chitosan-MnO2 nanocomposite for effective removal of cr (VI) from aqueous solution. *Chemosphere* **257**10.1016/j.chemosphere.2020.127147 (2020). 10.1016/j.chemosphere.2020.127147 32473410

[42] Morsy, M. et al. Synthesis and characterization of freeze dryer Chitosan nanoparticles as multifunctional eco-friendly finish for fabricating easy care and antibacterial cotton textiles. *Egypt. J. Chem.* **62** (7), 1277–1293. 10.21608/EJCHEM.2019.6995.1583 (2019).

[43] Kim, J. M., Huh, Y. S., Han, Y. K., Cho, M. S. & Kim, H. J. Facile synthesis route to highly crystalline mesoporous γ-MnO 2 nanospheres. *Electrochem. Commun.* **14** (1), 32–35. 10.1016/j.elecom.2011.10.023 (2012).

[44] Devi, R. et al. Electrochemical analysis of MnO2 (α, β, and γ)-Based electrode for High-Performance supercapacitor application. *Appl. Sci.* **13** (13). 10.3390/app13137907 (2023).

[45] Kim, S. T., Saha, K., Kim, C. & Rotello, V. M. The role of surface functionality in determining nanoparticle cytotoxicity. *Acc. Chem. Res.* **46** (3), 681–691. 10.1021/ar3000647 (2013). 23294365 10.1021/ar3000647 PMC3640732

[46] Hong, F. et al. Chitosan-based hydrogels: from Preparation to applications, a review. *Food Chem. X*. **21**, 101095. 10.1016/j.fochx.2023.101095 (2024). 38268840 10.1016/j.fochx.2023.101095 PMC10805631

[47] Queiroz, M. F., Melo, K. R. T., Sabry, D. A., Sassaki, G. L. & Rocha, H. A. O. Does the use of Chitosan contribute to oxalate kidney stone formation? *Mar. Drugs*. **13** (1), 141–158. 10.3390/md13010141 (2015). 10.3390/md13010141 PMC4306929 25551781

[48] Rostami, R. & Faraji, M. Porous MnO2–CNTs–Cellophane nanocomposite for High-Voltage flexible supercapacitors. *J. Inorg. Organomet. Polym. Mater.* **30** (9), 3438–3447. 10.1007/s10904-020-01546-1 (2020).

[49] Cotchim, S., Thavarungkul, P. & Kanatharana, P. Analytica chimica acta multiplexed label-free electrochemical immunosensor for breast cancer precision medicine. *Anal. Chim. Acta*. **1130**, 60–71. 10.1016/j.aca.2020.07.021 (2020). 32892939 10.1016/j.aca.2020.07.021

[50] Feng, J. et al. A novel sandwich-type electrochemical immunosensor for PSA detection based on PtCu bimetallic hybrid (2D/2D) rGO/g-C3N4. *Biosens. Bioelectron.* **91**, 441–448 (2017). 28064129 10.1016/j.bios.2016.12.070

[51] Choosang, J., Khumngern, S., Thavarungkul, P., Kanatharana, P. & Numnuam, A. An ultrasensitive label-free electrochemical immunosensor based on 3D porous chitosan–graphene–ionic liquid–ferrocene nanocomposite cryogel decorated with gold nanoparticles for prostate-specific antigen. *Talanta* **224**, 121787. 10.1016/j.talanta.2020.121787 (2021). 33379016 10.1016/j.talanta.2020.121787

[52] Ekwujuru, E. U. et al. Electrochemical and photoelectrochemical immunosensors for the detection of ovarian cancer biomarkers. *Sensors* **23** (8). 10.3390/s23084106 (2023). 10.3390/s23084106 PMC10142013 37112447

[53] Mistry, K. K., Layek, K., Mahapatra, A., RoyChaudhuri, C. & Saha, H. A review on amperometric-type immunosensors based on screen-printed electrodes. *Analyst* **139** (10), 2289–2311. 10.1039/c3an02050a (2014). 24678518 10.1039/c3an02050a

[54] He, X., Yuan, R., Chai, Y. & Shi, Y. A sensitive amperometric immunosensor for carcinoembryonic antigen detection with porous Nanogold film and nano-Au/chitosan composite as immobilization matrix. *J. Biochem. Biophys. Methods*. **70** (6), 823–829. 10.1016/j.jbbm.2007.06.002 (2008). 17669503 10.1016/j.jbbm.2007.06.002

[55] Gao, X., Zhang, Y., Chen, H., Chen, Z. & Lin, X. Amperometric immunosensor for carcinoembryonic antigen detection with carbon nanotube-based film decorated with gold nanoclusters. *Anal. Biochem.* **414** (1), 70–76. 10.1016/j.ab.2011.03.005 (2011). 21396907 10.1016/j.ab.2011.03.005

[56] Wang, X. et al. An ultrasensitive luminol cathodic electrochemiluminescence probe with highly porous Pt on ionic liquid functionalized graphene film as platform for carcinoembryonic antigen sensing. *Biosens. Bioelectron.* **141**10.1016/j.bios.2019.111436 (2019). 10.1016/j.bios.2019.111436 31226604

[57] Khan, M. S., Ameer, H. & Chi, Y. Label-Free and ultrasensitive electrochemiluminescent immunosensor based on novel luminophores of Ce2Sn2O7Nanocubes. *Anal. Chem.* **93** (7), 3618–3625. 10.1021/acs.analchem.0c05315 (2021). 33560834 10.1021/acs.analchem.0c05315

[58] Zhang, A. et al. Electrochemiluminescence immunosensor for sensitive determination of tumor biomarker CEA based on multifunctionalized Flower-like au@bsa nanoparticles. *Sens. Actuators B Chem.* **238**, 24–31. 10.1016/j.snb.2016.07.009 (2017).

[59] Zhao, L. et al. A novel ECL sensor for determination of carcinoembryonic antigen using reduced graphene Oxide-BaYF5:Yb, Er upconversion nanocomposites and gold nanoparticles. *Sens. Actuators B Chem.* **232**, 484–491. 10.1016/j.snb.2016.03.153 (2016).

[60] Jing, A., Xu, Q., Feng, W. & Liang, G. An electrochemical immunosensor for sensitive detection of the tumor marker carcinoembryonic antigen (CEA) based on three-dimensional porous nanoplatinum/graphene. *Micromachines* **11** (7). 10.3390/mi11070660 (2020). 10.3390/mi11070660 PMC7407820 32635249

[61] Cao, L. et al. A Label-Free electrochemical immunosensor for CEA detection on a novel signal amplification platform of Cu2S/Pd/CuO nanocomposites. *Front. Bioeng. Biotechnol.* **9** (December), 1–9. 10.3389/fbioe.2021.767717 (2021). 10.3389/fbioe.2021.767717 PMC8702859 34957069

[62] Jia, X., Chen, X., Han, J., Ma, J. & Ma, Z. Triple signal amplification using gold nanoparticles, bienzyme and platinum nanoparticles functionalized graphene as enhancers for simultaneous multiple electrochemical immunoassay. *Biosens. Bioelectron.* **53**, 65–70. 10.1016/j.bios.2013.09.021 (2014). 24113435 10.1016/j.bios.2013.09.021

[63] Yang, Y. et al. A novel label-free electrochemical immunosensor based on functionalized nitrogen-doped graphene quantum Dots for carcinoembryonic antigen detection. *Biosens. Bioelectron.* **90**, 31–38. 10.1016/j.bios.2016.11.029 (2017). 27871047 10.1016/j.bios.2016.11.029

[64] Jozghorbani, M., Fathi, M., Kazemi, S. H. & Alinejadian, N. Determination of carcinoembryonic antigen as a tumor marker using a novel graphene-based label-free electrochemical immunosensor. *Anal. Biochem.* **613**10.1016/j.ab.2020.114017 (2021). 10.1016/j.ab.2020.114017 33212021

[65] Dokur, E., Uruc, S., Gorduk, O. & Sahin, Y. Ultrasensitive electrochemical detection of carcinoembryonic antigen with a label-free immunosensor using gold nanoparticle-decorated poly(pyrrole-co-3,4-ethylenedioxythiophene). *ChemElectroChem* **9** (15). 10.1002/celc.202200121 (2022).

[66] Kong, F. Y., Xu, M. T., Xu, J. J. & Chen, H. Y. A novel lable-free electrochemical immunosensor for carcinoembryonic antigen based on gold nanoparticles-thionine-reduced graphene oxide nanocomposite film modified glassy carbon electrode. *Talanta* **85** (5), 2620–2625. 10.1016/j.talanta.2011.08.028 (2011). 21962692 10.1016/j.talanta.2011.08.028

[67] Gao, Y. S. et al. A label-free electrochemical immunosensor for carcinoembryonic antigen detection on a graphene platform doped with poly(3,4-ethylenedioxythiophene)/Au nanoparticles. *RSC Adv.* **5** (106), 86910–86918. 10.1039/c5ra16618g (2015).

[68] Feng, D., Lu, X., Dong, X., Ling, Y. & Zhang, Y. Label-free electrochemical immunosensor for the carcinoembryonic antigen using a glassy carbon electrode modified with electrodeposited Prussian blue, a graphene and carbon nanotube assembly and an antibody immobilized on gold nanoparticles. *Microchim. Acta*. **180** (9–10), 767–774. 10.1007/s00604-013-0985-8 (2013).

[69] Gao, Y. S. et al. Label-free electrochemical immunosensor based on nile blue A-reduced graphene oxide nanocomposites for carcinoembryonic antigen detection. *Anal. Biochem.* **500**, 80–87. 10.1016/j.ab.2016.02.010 (2016). 26898304 10.1016/j.ab.2016.02.010

