# Supplementary material for: A novel electrochemical immunosensor based on biomaterials for detecting carcinoembryonic antigen biomarker in serum samples
Source: Sci Rep. 2025 Jul 14;15:25396. doi: 10.1038/s41598-025-09547-1 (PMC12259841; doi:10.1038/s41598-025-09547-1)
Supplement: Supplementary file 1 — Supplementary Material 1 [file 41598_2025_9547_MOESM1_ESM.docx]

**A Novel Label-Free Electrochemical Immunosensor with Layer-by-Layer Modification of a Glassy Carbon Electrode Using Polysaccharide Biomaterials for Carcinoembryonic Antigen Detection in Serum Samples**

Sajedeh Sobhanparast^a,b^, Payam Shahbazi-Derakhshi^c^, Jafar Soleymani^d,^*, Amir Amiri Sadeghan^b^, Alireza Herischi^b^, Nader Chaparzadeh^a^, Younes Aftabi^b,^*

^a^ Department of Biology, Azarbaijan Shahid Madani University, Tabriz, Iran

^b^ Tuberculosis and Lung Diseases Research Center, Tabriz University of Medical Sciences, Tabriz, Iran

^c^ Liver and Gastrointestinal Diseases Research Center, Tabriz University of Medical Sciences, Tabriz, Iran

^d^ Pharmaceutical Analysis Research Center, Tabriz University of Medical Sciences, Tabriz, Iran

Co-corespondents: [jsoleymanii@gmail.com](mailto:jsoleymanii@gmail.com) and [soleymanij@tbzmed.ac.ir](mailto:soleymanij@tbzmed.ac.ir); [aftabiy@tbzmed.ac.ir](mailto:aftabiy@tbzmed.ac.ir)

# Figures

**Fig. 1S. BET/BJH plots.** Adsorption-desorption isotherm of BET surface area analysis (**A**), BET plot (**B**), and BJH plot (**C**) of γ.MnO_2_-CS.

**
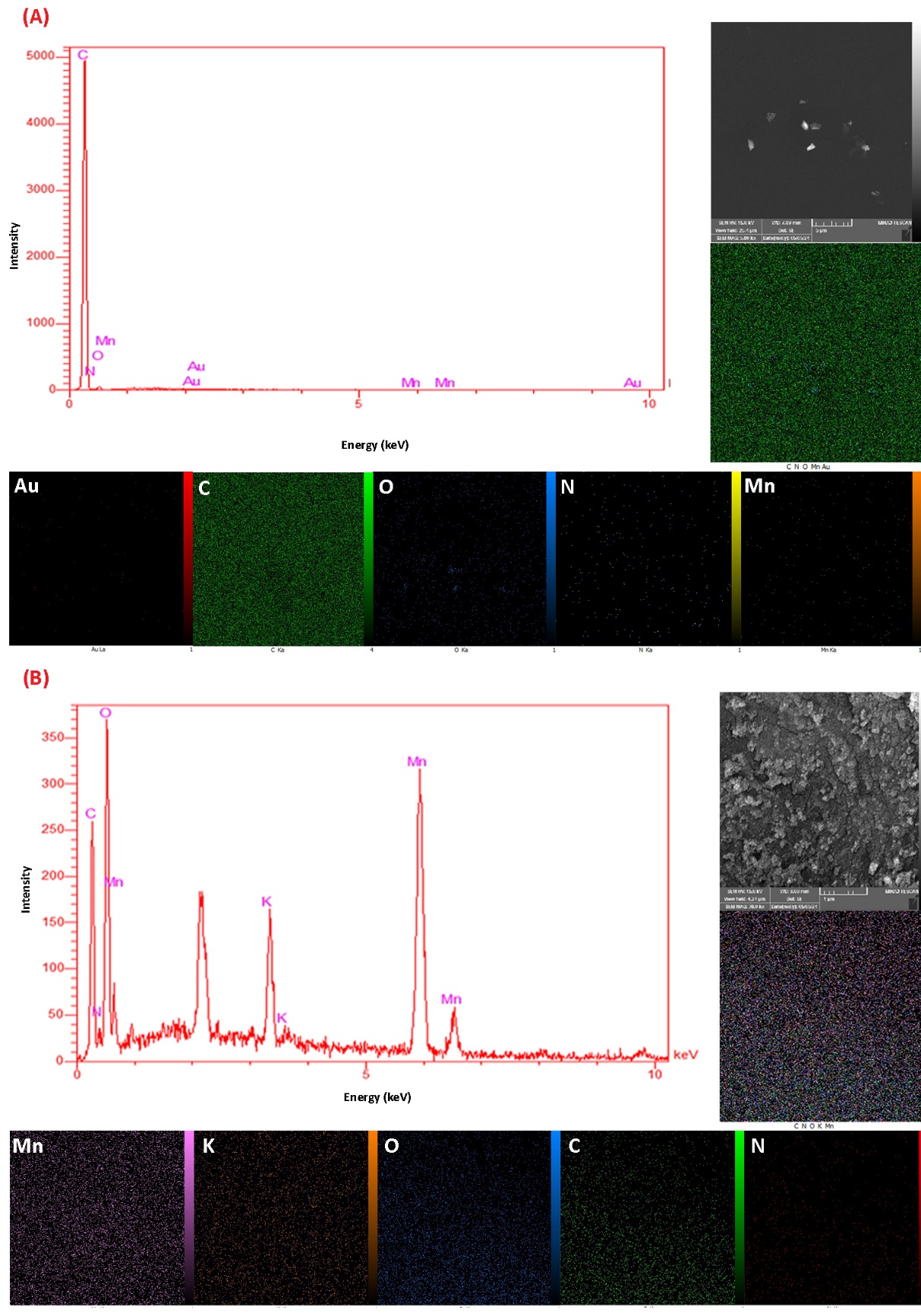
**

**Fig. 2S. EDX spectra.** EDX spectra of γ.MnO_2_-CS/AuNPs/SA/GCE (**A**) and γ.MnO_2_-CS (**B**).

| (**A**) |
| --- |
| 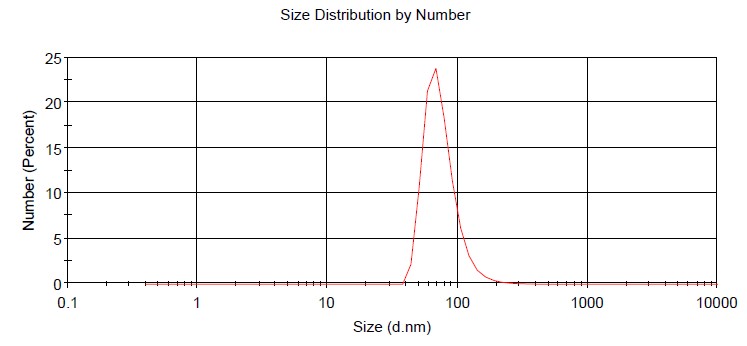 |
| (**B**) |
| 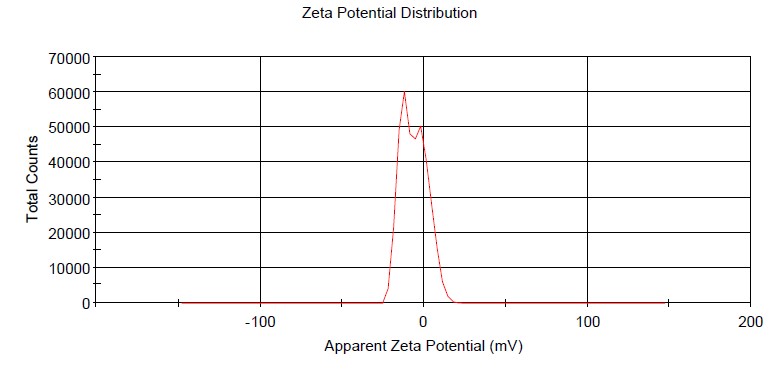 |
| (**C**) |
| 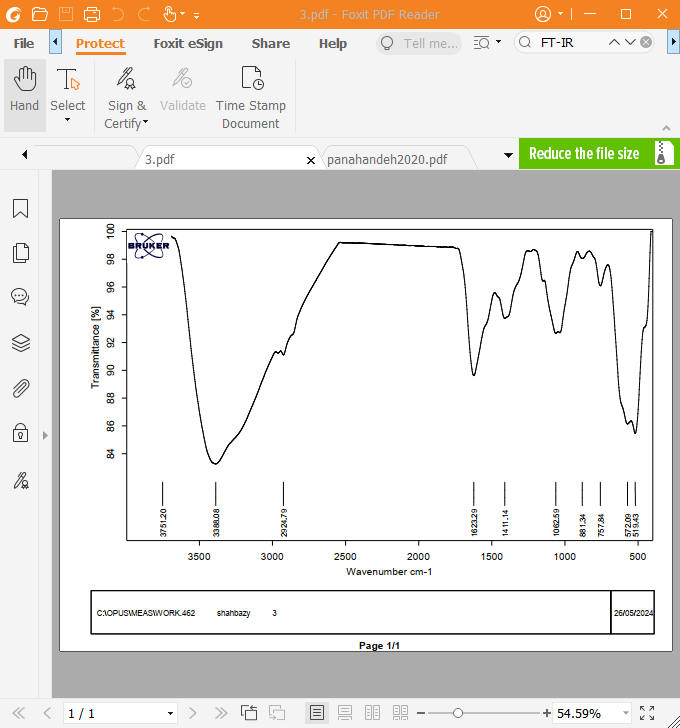 |

**Fig. 3S. DLS histogram (A), zeta potential (B), and FT-IR image of γ.MnO_2_-CS particles (C).**

(A)


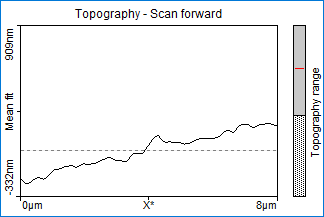

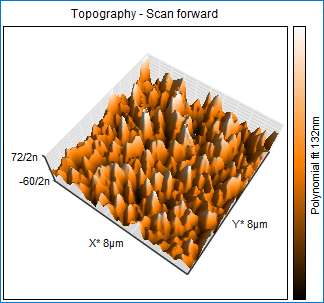

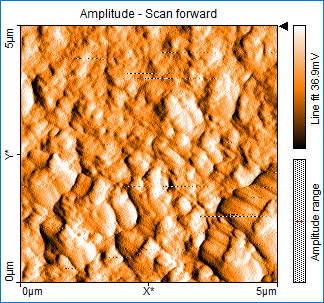


**Fig. 4S. AFM image of γ.MnO_2_-CS nanocomposite.**

| 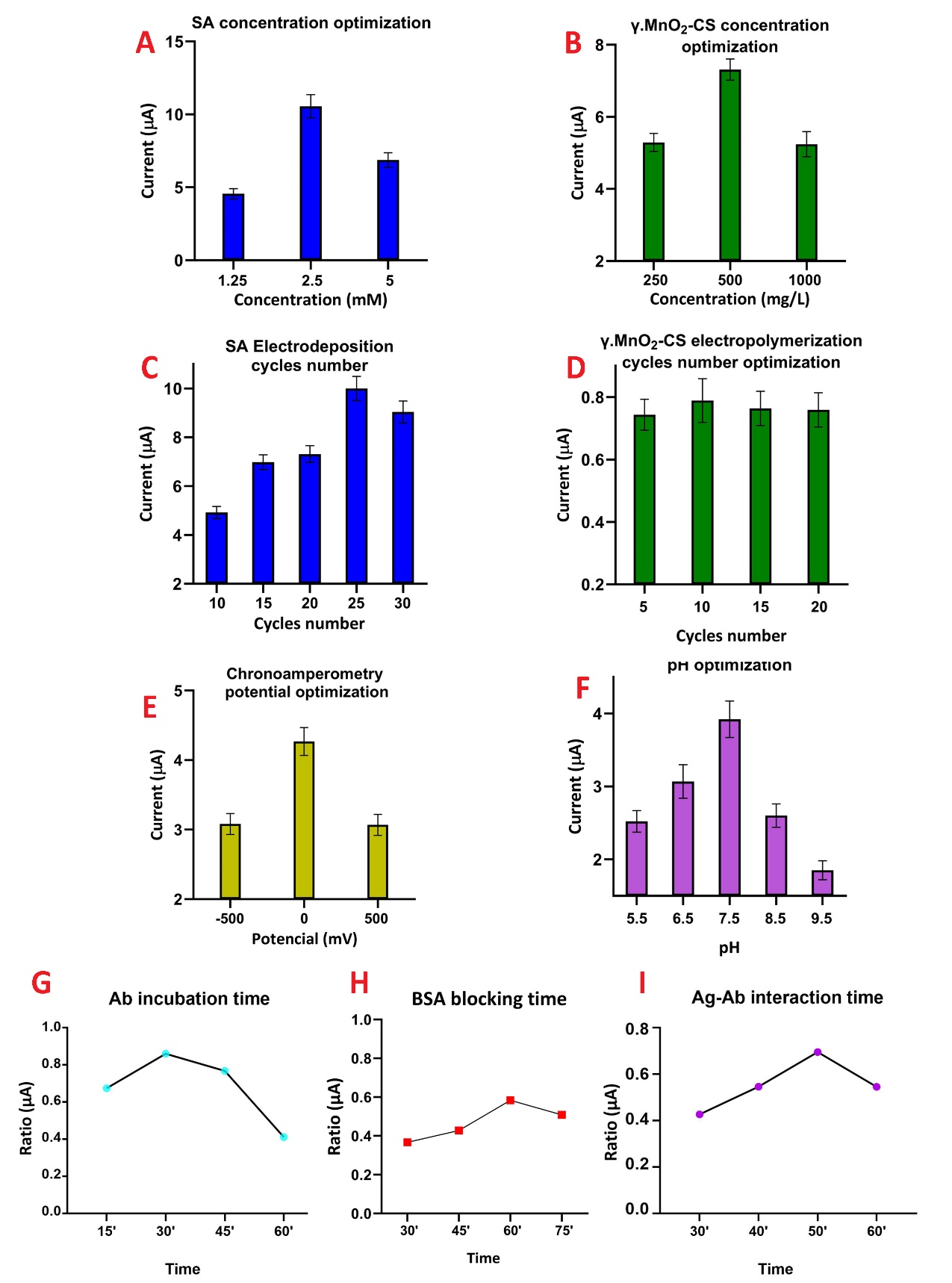 |
| --- |

**Fig. 5S. Optimization results.**Optimization of SA (**A**), and γ.MnO_2_-CS concentrations (**B**) SA (**C**) and γ.MnO_2_-CS electrodeposition cycle numbers (**D**). optimization of AuNPs chronoamperometry potential (**E**), and pH of redox probe ([Fe (CN)_6_]^3–/4–^) (**F**). Optimization of the main experimental factors: incubation time of Ab (**G**), blocking time (**H**) and incubation time of CEA-Ab with CEA (**I**).

**
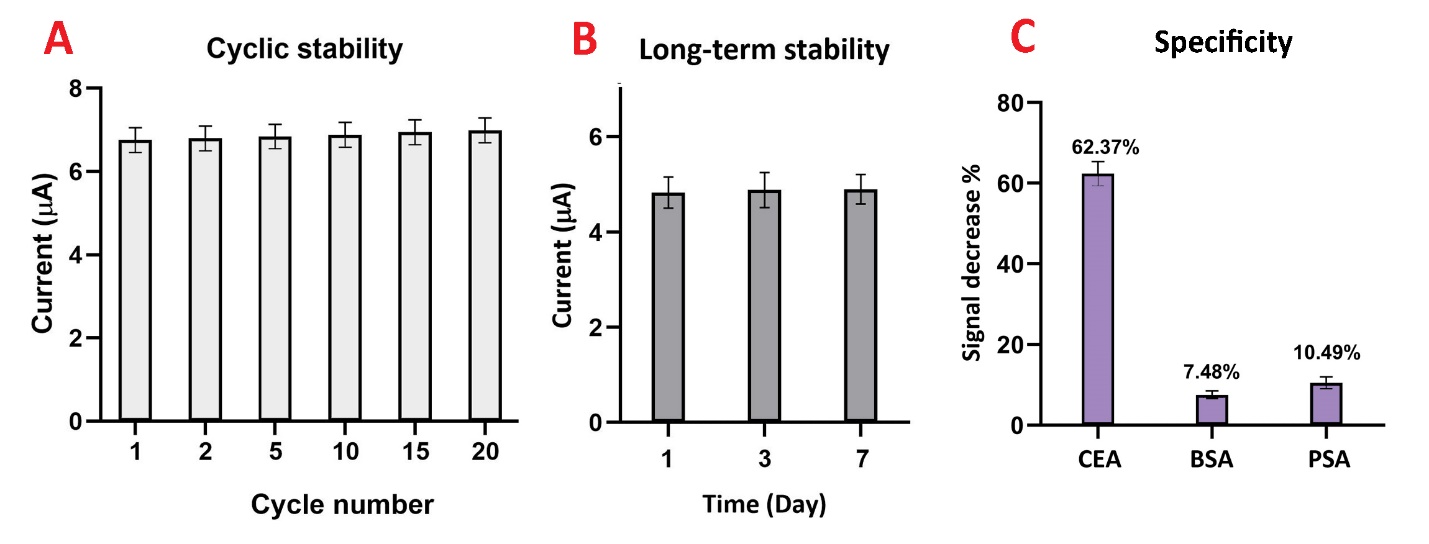
**

**Fig. 6S.** **Stability and specificity of the immunosensor.** stability of modified GCE in different cycle numbers (**A**), and long-term stability (**B**). Comparison of blank DPV signal reduction in different analytes: CEA (1ng/mL), BSA (15ng/mL), PSA (1.0 ng/mL) (**C**).

**Table S1. BET and BJH analysis results of γ.MnO₂-CS.**

| **A)BET analysis** |  |
| --- | --- |
| BET surface area | 30.055 m²/g |
| Total pore volume(*p*/*p*_0_=0.990) | 0.1941 cm³/g |
| Mean pore diameter | 25.834 nm |
| **B) BJH analysis** |  |
| BJH surface area | 36.761 m^2^/g |
| pore radius | 12.22 nm |
| total pore volume | 0.1961 cm³/g |
